# Blue magnetophosphene perception under subthreshold magnetic stimulation in a human retinal degeneration model

**DOI:** 10.3389/fnins.2026.1893022

**Published:** 2026-09-10

**Authors:** Omar Nawara, Eleonore Fresnel, Verena Juncal, Frank S. Prato, Cindy Hutnik, Nicolas Bouisset

**Affiliations:** 1 Department of Ophthalmology, Schulich School of Medicine and Dentistry, University of Western Ontario, London, ON, Canada; 2 Department of Medical Biophysics, University of Western Ontario, London, ON, Canada; 3 Lawson Research Institute, London, ON, Canada; 4 Department of Medical Imaging, University of Western Ontario, London, ON, Canada; 5 School of BioMedical Engineering, University of Western Ontario, London, ON, Canada; 6 EuroMov Digital Health in Motion, Université de Montpellier, IMT Mines Alès, Montpellier, France

**Keywords:** cortical perception, extremely low-frequency magnetic field (ELF-MF), magnetophosphenes, retinal modulation, transcranial magnetic stimulation (TMS), visual dysfunction

## Abstract

**Introduction:**

Extremely low-frequency magnetic fields (ELF-MF; <300 Hz) can induce visual percepts known as magnetophosphenes through weak, subthreshold electric fields in neural tissue. Although magnetophosphene perception has been characterized in healthy observers, the anatomical locus of interaction (retinal vs. cortical) remains incompletely constrained, particularly under conditions of severe retinal dysfunction.

**Methods:**

Here, we employed a controlled single-subject perturbation framework to mechanistically dissociate candidate sites of ELF-MF interaction using systematic variation of stimulation geometry, sham conditions, and independent suprathreshold transcranial magnetic stimulation (TMS) of the visual cortex.

**Results:**

ELF-MF exposure at 20 Hz and 100 millitesla root mean square (mT RMS) evoked reproducible, spatially uniform, blue-tinted visual percepts only when stimulation geometry favored ocular tissues, whereas occipital ELF-MF placement and all sham conditions produced no percept. In contrast, direct cortical stimulation with TMS generated qualitatively distinct, achromatic, retinotopically organized phosphenes and never reproduced the ELF-MF-evoked phenomenology.

**Discussion:**

Given the large disparity in induced electric field strengths between ELF-MF and TMS, these contrasts constrain plausible mechanisms to retinal or post-receptoral sites operating in a subthreshold modulation regime and argue against primary cortical initiation. While limited to a single constrained biological model, these findings refine current biophysical models of human magnetophosphene perception and motivate future studies combining replication, quantitative psychophysics, and individualized dosimetry to further delineate subthreshold electromagnetic interactions with the visual system.

## Introduction

Extremely low-frequency magnetic fields (ELF-MF; < 300 Hz) can evoke visual percepts known as magnetophosphenes through induction of weak electric fields in excitable neural tissue (Bouisset et al., 2020, 2025a; Legros et al., 2024). In humans, magnetophosphene perception exhibits a pronounced frequency dependence, with lowest perceptual thresholds occurring near 20 Hz (Bouisset et al., 2025a,b; Lövsund et al., 1980). At these frequencies and exposure levels, the induced electric fields are well below the threshold required for direct neuronal depolarization, supporting a mechanism based on subthreshold modulation of retinal networks rather than direct activation of visual cortex (Legros et al., 2024). Experimental and computational work has shown that ELF-MF exposure preferentially induces non-uniform electric fields within ocular tissues, with maxima in peripheral retinal regions and minimal cortical field strength, consistent with a retinal locus of interaction (Legros et al., 2024; Bouisset et al., 2025b).

Despite characterization of magnetophosphene thresholds and phenomenology in healthy observers, the anatomical origin of ELF-MF-evoked percepts remains incompletely constrained. In particular, it is unresolved whether such percepts require intact photoreceptor-driven retinal pathways or whether residual post-receptoral or inner-retinal circuitry is sufficient to generate conscious visual experience under subthreshold electromagnetic perturbation. Addressing this question is challenging using population-based designs, as magnetophosphene perception is variable and subjective, limiting the utility of averaging approaches.

Single-subject experimental designs offer an alternative strategy for mechanistic inference in such contexts (Holth, 2021; Krakauer et al., 2017). In sensory physiology and human neurostimulation research, intensive within-subject manipulation of stimulation parameters, anatomical targeting, and control conditions has long been used to test causal hypotheses when phenomena are rare, idiosyncratic, or non-averagable. In these designs, inference derives from systematic contrasts within a constrained biological model rather than from statistical prevalence across participants (Krakauer et al., 2017; Hallett, 2007). This approach is particularly well suited for interrogating the locus of action of weak electromagnetic stimulation, where induced field strengths, spatial distributions, and perceptual outcomes can be precisely controlled and compared.

Here, we apply this experimental logic to investigate the origin of magnetophosphene perception under conditions of severe retinal dysfunction. The experimental model is characterized by profound attenuation of classical photoreceptor-mediated retinal responses, as established by standard electrophysiological measures, providing a stringent constraint on candidate mechanisms. Within this model, ELF-MF exposure was applied under controlled conditions with systematic variation of coil placement to differentially target retinal vs. cortical structures, combined with sham stimulation to control for non-specific effects. To further dissociate retinal from cortical contributions, suprathreshold transcranial magnetic stimulation (TMS) of visual cortex was employed as an independent positive control for direct cortical phosphene generation (Kammer et al., 2005; Stewart et al., 2001). Positive control is defined herein as a condition in which specific known effects are expected (Johnson and Besselsen, 2002). This combination of perturbations permits exclusion-based causal inference. If ELF-MF-evoked percepts were mediated primarily by cortical activation, they would be expected to resemble or be reproduced by direct cortical stimulation at substantially higher induced electric field strengths. Conversely, percepts arising from retinal or post-receptoral mechanisms should depend on stimulation geometry favoring ocular tissues and exhibit phenomenological features distinct from canonical TMS-induced cortical phosphenes. By comparing perceptual outcomes across these conditions within the same biological model, the present study constrains the plausible anatomical locus of ELF-MF interaction without reliance on population-level statistics.

The goal of this work is therefore not to establish prevalence or clinical efficacy, but to mechanistically interrogate the capacity of residual visual circuitry to transduce weak electromagnetic fields into conscious percepts. By leveraging controlled within-subject contrasts, this study seeks to clarify the conditions under which magnetophosphene perception can arise in the near absence of classical photoreceptor-driven input and to refine current models of ELF-MF interaction with the human visual system.

## Methods

### Experimental design and rationale

This study was designed as a controlled single-subject mechanistic investigation employing repeated within-subject manipulations to test competing hypotheses regarding the origin of magnetophosphene perception. The experimental logic follows established principles in human sensory physiology and neurostimulation research, in which causal inference is derived from systematic condition contrasts rather than inter-individual variability (Holth, 2021; Krakauer et al., 2017; Hallett, 2007). Accordingly, the participant was treated as a biological model, and perceptual outcomes were evaluated as a function of stimulation parameters, anatomical targeting, and modality.

The primary mechanistic question was whether ELF-MF-evoked visual percepts arise from retinal subthreshold modulation or from direct cortical activation. To address this, stimulation conditions were selected to differentially engage these pathways while holding other variables constant. ELF-MF exposure was applied at periorbital and frontal midline locations to preferentially target retinal tissues, and at the occipital region to control for potential cortical induction. Sham stimulation was included to control for expectancy and non-specific effects. In a separate session, transcranial magnetic stimulation (TMS) was delivered to the visual cortex at suprathreshold intensities to provide a positive control for cortical phosphene generation.

### Participant as experimental model

The participant's advanced Stargardt disease (see Supplementary material), characterized by profound photoreceptor dysfunction and severely attenuated full-field and multifocal electroretinographic responses (Supplementary Figures S1–S3), constituted a constrained biological model in which canonical rod-mediated mechanisms of magnetophosphene perception would be expected to be minimal. This pathology therefore served not as a clinical endpoint, but as an experimental constraint, enabling mechanistic interrogation of residual post-receptoral and inner-retinal circuitry under controlled stimulation conditions. All perceptual reports were collected within the same individual across conditions, allowing direct comparison of phenomenology under distinct causal manipulations.

### Ethical considerations and experimental control

All procedures were conducted in accordance with the Declaration of Helsinki and were approved by Western University's Health Sciences Research Ethics Board (no. 109344). Written informed consent was obtained prior to participation. To minimize confounding factors, the participant was naïve to brain stimulation paradigms and underwent standardized screening for contraindications to magnetic and electrical stimulation (Antal et al., 2017; Rossi et al., 2021). Experimental sessions were conducted under controlled lighting conditions, with systematic dark adaptation periods (Bouisset et al., 2025a) and inter-trial intervals to reduce adaptation, fatigue, and carryover effects.

### Experimental device

The ELF-MF coil used in this study is the same system that has already been employed in several other human experiments (Bouisset et al., 2025a; Legros et al., 2024): the same tAMS single coil geometry and retinal and occipital placement protocols were previously evaluated in healthy participants (Legros et al., 2024; Fresnel et al., 2026). Retinal tAMS produced a robust intensity-dependent increase in magnetophosphene perception, whereas occipital stimulation yielded only weak perceptual responses and substantially higher estimated perceptual thresholds. Computational dosimetry using the same coil geometry showed that the two placements generated comparable maximum *in situ* electric-field magnitudes of approximately 0.3 V/m per 1 T/s, but with different spatial distributions. Occipital placement preferentially exposed the posterior cortical regions, whereas retinal placement preferentially exposed the retina and frontal/temporal regions. Using the same coil configuration ensures methodological consistency with established magnetophosphene research and supports reliable comparison with previously reported findings.

### Experimental procedure

After providing informed consent, the participant completed two experimental sessions: the first lasting 1.5 h and the second lasting 30 min.

Exclusion criteria included any history of claustrophobia, head injury, neurological or cardiovascular disease, history of epilepsy or seizures, as well as the presence of permanent metallic implants above the neck or any implanted electronic devices, including neural stimulators, cardiac pacemakers, defibrillators, cochlear implants, or insulin pumps.

To minimize potential physiological and perceptual confounds, the participant was instructed to abstain from strenuous physical activity and to avoid alcohol, caffeine, nicotine, and all pharmaceutical or recreational drugs for at least 24 h before the session (Antal et al., 2017). All procedures were conducted under medical supervision at the Bioelectromagnetic Research Facility, St. Joseph's Hospital (London, Ontario, Canada), in accordance with established safety standards for non-invasive brain and sensory stimulations (Antal et al., 2017).

In the first session, for each of the 12 stimulations epochs, the participant received exposures at 100 mT_RMS_ at 20 Hz. This was chosen to increase the probability of inducing phosphene perception for the patient as it corresponds to 100% probability of magnetophosphene perception in participants with healthy visual systems (Legros et al., 2024; Fresnel et al., 2026).

**Figure 1:**
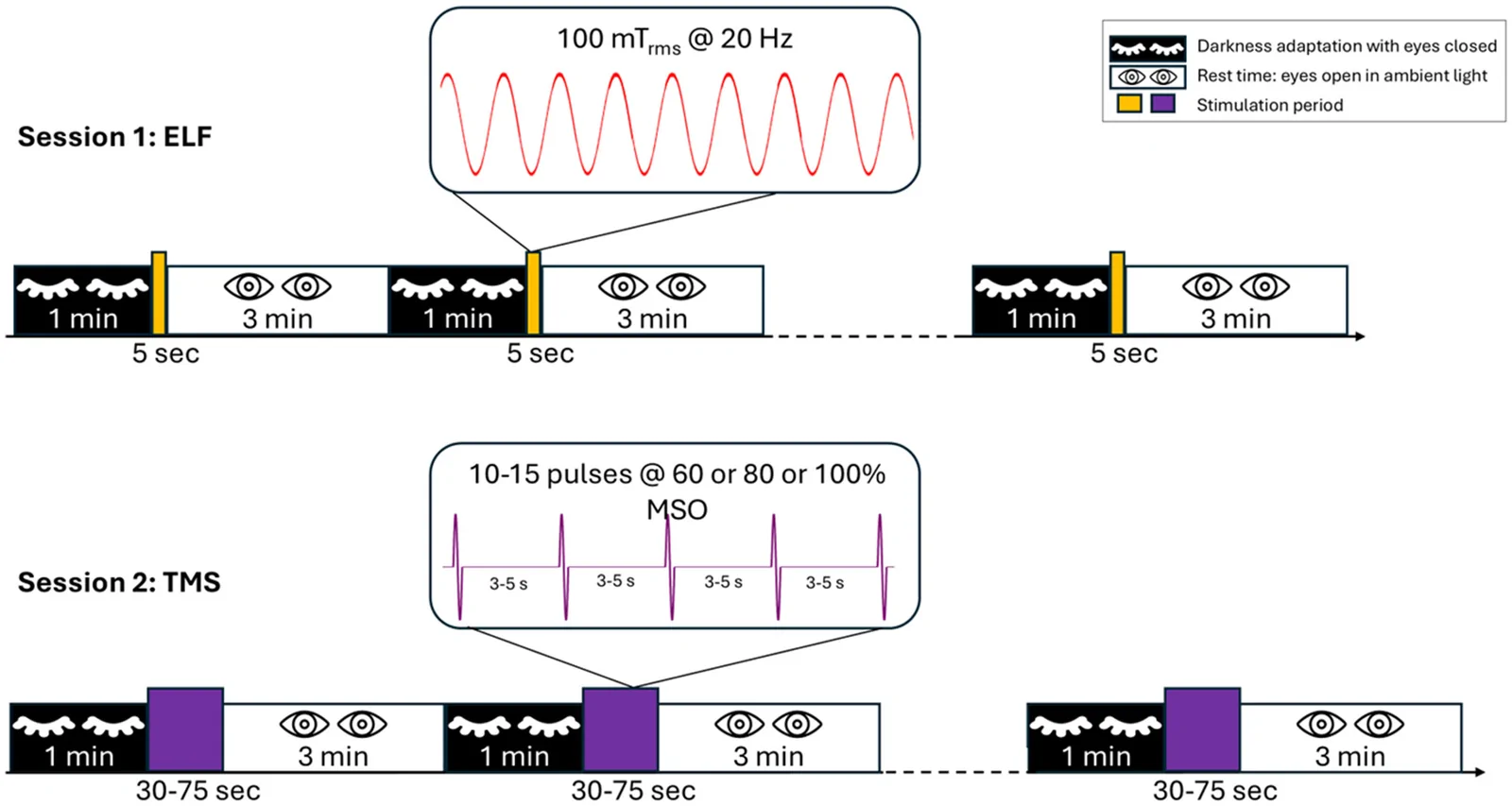
Schematic overview of the experimental stimulation protocols. The ELF-MF protocol (**top**) consisted of repeated 5-s exposure trials at 20 Hz and 100 mT_RMS_, separated by 3-min rest intervals, with a 1-min dark adaptation period preceding each block. Coil placement was systematically varied to target periorbital, frontal midline, or occipital regions, and sham conditions were interleaved. The TMS protocol **(bottom)** involved stimulation of the visual cortex at three intensities (60%, 80%, and 100% of maximum stimulator output). For each intensity-hemisphere condition, 10–15 pulses were delivered with 3–5 s inter-pulse intervals, followed by a 3-min rest period. Each condition was preceded by 1 min of dark adaptation. Participant reports were collected after each trial or pulse.

The participant sat comfortably in complete darkness during the testing epochs. To achieve a stable baseline of retinal sensitivity while maintaining experimental efficiency, a 1-min dark adaptation period was implemented prior to each exposure trial (Legros et al., 2024). Although complete rod-mediated dark adaptation requires substantially longer durations, short intervals are sufficient to stabilize cone-mediated responses and attenuate residual effects of prior light exposure (Bouisset et al., 2025a). This duration thus represented a physiologically optimal compromise, providing consistent luminance and contrast sensitivity while preventing the confounding influence of prolonged rod activation. Each MF stimulation was tested across 5-s exposure trials, separated by 3-min rest intervals. The 3-min rest was chosen primarily to allow the participant to describe the perceived magnetophosphenes in detail and for these descriptions to be documented, and also to prevent fatigue, enable dissipation of any transient stimulation effects, and minimize carryover between trials (Figure 1). This interval also provided a standardized separation between conditions but was not intended to reflect an established physiological recovery time constant. The lights were switched back on right after the stimulation was finished. After each trial, the participant was instructed to report and describe any perceived visual experience.

**Figure 2:**
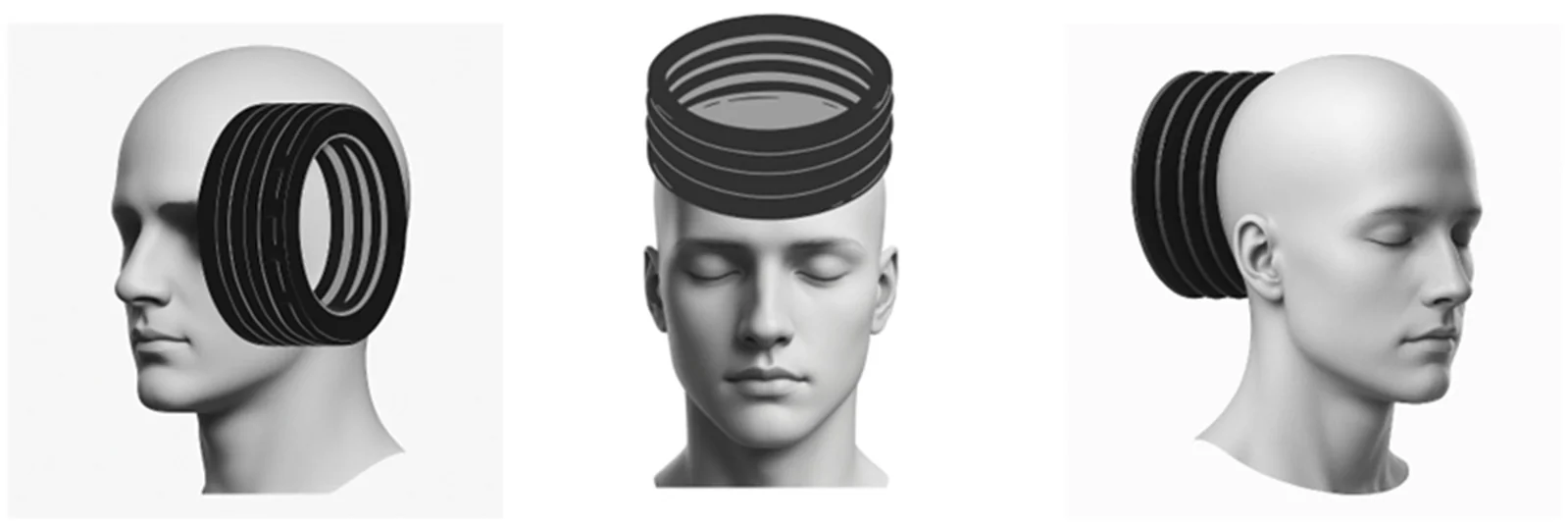
Schematic illustration of exposure coil positioning for magnetophosphene induction. **Left**: Extremely low frequency (ELF) magnetic stimulation coil centered over the eye to target the retina. Middle: Coil positioned over the vertex of the head (Frontal midline). **Right**: Coil centered over the occipital region to stimulate the visual cortex. The figure was generated using OpenAI's image generation tool integrated within ChatGPT (November 2025 version- Available at: https://openai.com/chatgpt).

Control conditions were implemented to verify the specificity of reported percept: (Bouisset et al., 2020) using an identical coil configuration with no current applied; (Bouisset et al., 2025a) as in a previous protocol in healthy participants, we relocated the active ELF coil over the occipital cortex to control for direct cortical activation (Figure 2 right panel) (Legros et al., 2024; Fresnel et al., 2026). The exposure conditions were administered in the following fixed order: right-eye, frontal midline, occipital, and left-eye stimulation. Within each condition, sham trials were presented in a randomized order.

In the second session, we delivered single-pulse TMS to the visual cortex using a figure-of-eight D70^2^ coil connected to a Magstim Rapid^2^ Plus^1^ stimulator (Magstim Company Ltd., Whitland, UK). Stimulation of visual cortical areas with TMS is known to induce achromatic phosphenes, whose perceived location typically corresponds to the retinotopic representation of the stimulated cortical site (Graaf et al., 2014). The participant sat comfortably and was exposed to three stimulation intensities (60%, 80%, and 100% of maximum stimulator output [MSO]) applied over both the left and right occipital cortex.

After 1 min in the dark, each intensity-hemisphere condition consisted of 10 to 15 single pulses, delivered with a 3–5 s inter-pulse interval to minimize adaptation or carry-over effects. For each pulse, the participant was asked to report the presence or absence of any percept, as well as the location and qualitative characteristics of any evoked phosphenes. Each intensity-hemisphere condition was separated by 3-min rest intervals (Figure 1).

## Results

### ELF-exposures

Active exposures at periorbital sites and at frontal midline (Figure 2: Left and middle panels) consistently evoked a large luminous band, whereas occipital placement (Figure 2: right panel) and all sham conditions produced no percept. With right-eye placement, straight-ahead fixation yielded an arcuate band spanning roughly 150° of visual angle; horizontal gaze reproduced the band, upward gaze increased apparent brightness, and downward gaze produced similar or slightly greater intensity. The reported visual angles were approximate subjective estimates. The participant indicated the perceived extent of the phosphene verbally.

With left-eye placement, the band remained present with a sharper ipsilateral contour and mild left-right asymmetry across gaze positions; in contralateral gaze the percept terminated immediately at stimulus offset, while other conditions showed a brief persistence of 1–2 s. The participant described the induced band as “blue”, for each magnetophosphenes perception. Table 1 summarizes stimulation-response pairs for all coil positions and gaze directions.

**Table 1:** Summary of stimulation conditions and perceptual responses.

Coil position	Gaze direction	Percept description	Relative brightness	Persistence after stimulation
Occipital	Straight	None (faint afterimage only)	None	None
Right eye	Straight	Broad arcuate band (~150°)	Moderate	None
	Right	Same band, same pattern	Moderate	None
	Left	Same band, same intensity and pattern	Moderate	None
	Up	Same band, increased brightness	Slightly higher	None
	Down	Same band, comparable intensity	Similar or slightly higher	None
Frontal midline	Straight	Broad band, similar to right-eye stimulation	Moderate	None
Left eye	Straight	Broad band, sharper on stimulated side	Moderate	None
	Right	Same band, immediate cessation at offset	Moderate	None
	Left	Same band, mild asymmetry	Moderate	Brief (1–2 s)
	Up	Band less bright, less uniform	Lower	None
	Down	Band brighter than up gaze	Higher	None

### TMS control

Stimulation at 60% MSO did not evoke any percepts in either hemisphere. At 80% MSO, single-pulse TMS reliably induced brief achromatic percepts described by the participant as “white points” or “flashes.” These phosphenes were perceived primarily unilaterally and were subjectively more intense in the visual field corresponding to the stimulated hemisphere (i.e., right-hemisphere stimulation produced stronger percepts in the right visual field and left-hemisphere stimulation in the left). Perceptions were stronger and more consistent on the right when the right hemisphere was stimulated, and noticeably weaker on the left during left-hemisphere stimulation. At 100% MSO, the participant reported percepts that were qualitatively similar to those at 80%, but stronger in intensity and characterized by a flatter, more homogeneous pattern, while the hemispheric lateralization of percept strength remained evident.

## Discussion

The present study employed a controlled single-subject perturbation framework to constrain the anatomical locus of ELF-MF-evoked visual percepts under conditions of severe retinal dysfunction. By systematically varying stimulation geometry, including periorbital, frontal, and occipital ELF-MF exposure, incorporating sham conditions, and contrasting these effects with suprathreshold TMS of visual cortex, we implemented an exclusion-based logic that does not rely on population-level statistics. Within this constrained biological model, ELF-MF exposure produced reproducible visual percepts only when stimulation geometry favored ocular tissues and not when cortical structures were preferentially targeted. Importantly, direct cortical stimulation at electric field strengths orders of magnitude greater than those induced by ELF-MF generated qualitatively distinct percepts, consistent with canonical cortical phosphenes and never reproduced the ELF-MF-evoked phenomenology. Taken together, these contrasts constrain plausible mechanisms to retinal or post-retinal sites of interaction and argue against a primary cortical origin for the observed effects.

A critical constraint on interpretation is imposed by the relative magnitudes and spatial distributions of electric fields induced by ELF-MF and transcranial magnetic stimulation. At 20 Hz and 100 mT_RMS_, ELF-MF exposure induces *in situ* electric fields on the order of 0.1–0.5 V/m within retinal tissues, corresponding to current densities well below those required to directly elicit neuronal depolarization (Legros et al., 2024). In contrast, single-pulse TMS over visual cortex produces transient cortical electric fields typically exceeding 50–100 V/m, depending on coil geometry and stimulator output, and is known to reliably evoke action potentials in cortical neurons. These values differ by two to three orders of magnitude, placing ELF-MF firmly within a subthreshold modulation regime that is biophysically incompatible with direct cortical excitation (Legros et al., 2024).

Importantly, subthreshold electric fields of the magnitude induced by ELF-MF are insufficient to drive cortical neurons to firing threshold but can modulate membrane potentials, synaptic efficacy, and network excitability in spatially extended structures (Radman et al., 2009; Liu et al., 2018). In the cortex, such weak fields are further attenuated by distance from the stimulation source and by the orientation and laminar organization of pyramidal neurons, rendering direct cortical involvement implausible under the present exposure conditions. By contrast, ocular tissues are positioned in close proximity to the ELF-MF source during periorbital stimulation, and modeling studies consistently predict substantially higher and more spatially structured field distributions within the retina than within occipital cortex under identical exposure parameters (Legros et al., 2024). This arises from the close proximity of ocular tissues to the stimulation source, the smaller characteristic dimensions of the eye, and the strong conductivity contrasts at tissue boundaries, all of which amplify local electric field gradients. Because the induced current density scales with the spatial derivative of the electric field and with tissue conductivity, these geometric and biophysical factors lead to proportionally larger local current densities within retinal layers than in deeper cortical structures. In contrast, cortical tissues experience greater attenuation due to distance from the coil, skull and cerebrospinal fluid filtering, and more homogeneous volume conduction, resulting in weaker and more diffuse induced currents.

The contrast with TMS therefore provides a decisive test of cortical involvement. If ELF-MF-evoked percepts arose from cortical mechanisms, then direct cortical stimulation at electric field strengths orders of magnitude greater should have evoked percepts that were at minimum comparable in spatial extent and phenomenology. Instead, TMS reliably produced brief, achromatic, retinotopically organized phosphenes consistent with established cortical activation (Hallett, 2007; Ca and Walsh, 2000; Fried et al., 2011) and never reproduced the ELF-MF-evoked percepts. This qualitative dissociation, combined with the large disparity in induced field strengths, strongly argues against a cortical origin and supports the interpretation that ELF-MF interacts with retinal or post-retinal circuitry operating in a subthreshold regime.

Given the biophysical constraints outlined above, a retinal or post-receptoral locus of interaction provides a parsimonious explanation for the observed perceptual effects. Although classical photoreceptor-driven responses were profoundly attenuated in this experimental model, residual inner-retinal and ganglion-cell circuitry is known to persist in advanced retinal degeneration and to remain electrically excitable even in the absence of robust outer-retinal input (Jones and Marc, 2005; Marc et al., 2003). Subthreshold electric fields of the magnitude induced by ELF-MF are sufficient to bias membrane potentials and synaptic gain within these networks, particularly in spatially extended retinal circuits with low temporal bandwidth, without necessitating action potential initiation (Bouisset et al., 2025a; Legros et al., 2024; Bouisset et al., 2025b). Under these conditions, weak electromagnetic perturbation could generate a sparse or biased afferent signal that is insufficient to convey spatial structure but capable of driving a consistent perceptual response.

The chromatic bias reported during ELF-MF exposure further supports involvement of post-receptoral retinal pathways rather than direct cortical activation. Color perception arises from differential activation of parallel retinal channels, and selective modulation of surviving pathways could bias perceptual output without requiring intact photoreceptor mosaics. Importantly, such bias need not reflect precise chromatic encoding, but rather a relative weighting of residual retinal signals that are subsequently interpreted by cortical color-processing mechanisms. In this framework, chromatic phenomenology reflects the spectral signature of the afferent drive rather than cortical generation of color *per se*.

The spatial uniformity of the percept is likewise consistent with a retinal initiation followed by cortical integration (De Weerd, 2006). A weak, spatially diffuse retinal signal lacking fine retinotopic structure would be expected to undergo completion and homogenization within early visual cortex, where sparse or ambiguous input is routinely transformed into coherent perceptual fields. Crucially, this integrative role of cortex does not imply cortical initiation of the percept (De Weerd, 2006). Instead, it reflects the transformation of minimal retinal input into a stable perceptual experience, a process well documented in conditions of degraded or absent visual information. Thus, uniformity of the percept is compatible with cortical processing of a retinally initiated signal and does not contradict a retinal or post-receptoral locus of primary interaction (De Weerd, 2006).

Several limitations inherent to the present study should be explicitly acknowledged. First, the experimental framework relies on a single-subject design, which precludes statistical inference and does not permit assessment of inter-individual variability. While this approach is appropriate for mechanistic exclusion in non-averagable phenomena, replication in additional constrained systems will be required to determine the generality of the observations. Second, perceptual reports were necessarily subjective and qualitative; no psychophysical thresholds, quantitative luminance measures, or chromatic matching procedures were employed. Consequently, the reported chromatic bias cannot be independently verified and should be interpreted as phenomenological rather than metrically precise. Third, although electrophysiological evidence indicates profound attenuation of classical photoreceptor-driven responses, the precise residual retinal pathways contributing to the observed percepts cannot be identified from the present data alone. Fourth, while the contrast with suprathreshold TMS strongly constrains cortical initiation, the present design cannot exclude a role for cortical processing in shaping or integrating the percept once retinal input is generated. Finally, the stimulation parameters were selected to maximize perceptual detectability rather than to map thresholds or dose-response relationships, limiting conclusions regarding sensitivity or optimal exposure conditions. These limitations underscore that the present findings constrain plausible mechanisms rather than establish definitive loci or pathways and motivate future studies incorporating replication, quantitative psychophysics, and subject-specific field modeling. Future studies combining subject-specific dosimetry with electrophysiological and functional neuroimaging approaches, such as MRI-compatible HD-EEG or whole-head fNIRS, could further refine the relative retinal and cortical contributions to ELF-MF-evoked percepts and explore whether consistent chromatic biases correspond to particular post-receptoral pathways.

## Conclusion

In summary, this study used a controlled single-subject perturbation framework to constrain the anatomical locus of ELF-MF-evoked visual percepts under conditions of severe retinal dysfunction. By combining systematic variation of stimulation geometry, sham controls, and direct suprathreshold cortical stimulation, the findings support a retinal or post-receptoral origin operating in a subthreshold modulation regime and argue against primary cortical initiation. The qualitative dissociation between ELF-MF-evoked percepts and canonical TMS-induced cortical phosphenes, together with established biophysical field constraints, provides converging evidence for this interpretation. While the present work does not establish definitive pathways or generalizability, it demonstrates that residual visual circuitry can transduce weak electromagnetic perturbation into conscious percepts even in the near absence of classical photoreceptor-driven input. These findings refine current models of human magnetophosphene perception and motivate future studies employing replication, quantitative psychophysics, and individualized dosimetry to further delineate the mechanisms underlying subthreshold electromagnetic interactions with the visual system. In the longer term, and pending replication and validation, such approaches might inform the assessment of residual visual pathway excitability in the context of emerging retinal restoration strategies.

## Data Availability

The original contributions presented in the study are included in the article/supplementary material, further inquiries can be directed to the corresponding author/s.

## References

[B1] Antal A. Alekseichuk I. Bikson M. Brockmöller J. Brunoni A. R. Chen R. . (2017). Low intensity transcranial electric stimulation: safety, ethical, legal regulatory and application guidelines. Clin. Neurophysiol. 128, 1774–1809. doi: 10.1016/j.clinph.2017.06.001 PMC5985830 28709880

[B2] Bouisset N. Carvallo A. Laporte M. Legros A. (2025b). Human achromatic flickers and phosphenes thresholds under extremely low frequency electric stimulations. Sci. Rep. 15:23779. doi: 10.1038/s41598-025-06271-8 40610500 PMC12226723

[B3] Bouisset N. Carvallo A. Villard S. Laakso I. Legros A. (2025a). Frequency responses of human magnetophosphene perception thresholds during dark adaptation point to rod modulation. Exp. Physiol. 111, 1242–1252. doi: 10.1113/EP092852 40973130 PMC12949185

[B4] Bouisset N. Villard S. Legros A. (2020). Human postural control under high levels of extremely low frequency magnetic fields. IEEE Access 8, 101377–101385. doi: 10.1109/ACCESS.2020.2997643

[B5] Ca A. C. Walsh V. (2000). Magnetically induced phosphenes in sighted, blind and blindsighted observers. Neuroreport. 11, 3269–3273. doi: 10.1097/00001756-200009280-00044 11043562

[B6] De Weerd P. (2006). Perceptual filling-in : more than the eye can see. Prog. Brain Res. 154, 227–245. doi: 10.1016/S0079-6123(06)54012-9 17010714

[B7] Fresnel E. Penault M. Moulin M. Blaquart J. Carvallo A. Bouisset N. (2026). Reproducible magnetophosphene thresholds induced by transcranial alternating magnetic stimulation in humans: a replication study. Sci. Rep. 16:14368. doi: 10.1038/s41598-026-44440-5 41865084 PMC13144530

[B8] Fried P. J. Elkin-Frankston S. Rushmore R. J. Hilgetag C. C. Valero A. (2011). Characterization of visual percepts evoked by noninvasive stimulation of the human posterior parietal cortex. PLoS ONE 6, 1–12. doi: 10.1371/journal.pone.0027204 22087266 PMC3210763

[B9] Graaf T. A. De Koivisto M. Jacobs C. Sack A. T. (2014). The chronometry of visual perception : review of occipital TMS masking studies. Neurosci. Biobehav. Rev. 45, 295–304. doi: 10.1016/j.neubiorev.2014.06.017 25010557

[B10] Hallett M. (2007). Transcranial magnetic stimulation : a primer. Neuron 55, 187–199. doi: 10.1016/j.neuron.2007.06.026 17640522

[B11] Holth P. (2021). A retrospective 60-year review of Murray Sidman' stactics of scientific research and some of its influence on behavior analysis. J. Exp. Anal. Behav. 115, 86–101. doi: 10.1002/jeab.645 33210290

[B12] Johnson P. Besselsen D. (2002). Practical aspects of experimental design in animal research experimental design : initial steps. Inst. Lab. Anim. Res. 43, 203–206. doi: 10.1093/ilar.43.4.202 12391395

[B13] Jones B. W. Marc R. E. (2005). Retinal remodeling during retinal degeneration. Exp. Eye Res. 81, 123–137. doi: 10.1016/j.exer.2005.03.006 15916760

[B14] Kammer T. Puls K. Erb M. (2005). Transcranial magnetic stimulation in the visual system. II. Characterization of induced phosphenes and scotomas. Exp. Brain Res. 160, 129–140. doi: 10.1007/s00221-004-1992-0 15368087

[B15] Krakauer J. W. Ghazanfar A. A. Gomez-Marin A. Maciver M. A. Poeppel D. (2017). Perspective neuroscience needs behavior : correcting a reductionist bias. Neuron 93, 480–490. doi: 10.1016/j.neuron.2016.12.041 28182904

[B16] Legros A. Nissi J. Laakso I. Duprez J. Kavet R. Modolo J. (2024). Thresholds and mechanisms of human magnetophosphene perception induced by low frequency sinusoidal magnetic fields. Brain Stimulat. 17, 668–675. doi: 10.1016/j.brs.2024.05.004 38740182

[B17] Liu A. Vöröslakos M. Kronberg G. Henin S. Krause M. R. Huang Y. (2018). Immediate neurophysiological effects of transcranial electrical stimulation. Nat. Commun. 9:5092. doi: 10.1038/s41467-018-07233-7 30504921 PMC6269428

[B18] Lövsund P. Öberg P. Å. Nilsson S. E. G. Reuter T. (1980). Magnetophosphenes: a quantitative analysis of thresholds. Med. Biol. Eng. Comput. 18, 326–334. doi: 10.1007/BF02443387 6968384

[B19] Marc R. E. Jones B. W. Watt C. B. Strettoi E. (2003). Neural remodeling in retinal degeneration. Prog. Retin. Eye Res. 22, 607–655. doi: 10.1016/S1350-9462(03)00039-9 12892644

[B20] Radman T. Ramos R. L. Brumberg J. C. Bikson M. (2009). Role of cortical cell type and morphology in subthreshold and suprathreshold uniform electric field stimulation in vitro. Brain Stimulat. 2, 215–228.e3. doi: 10.1016/j.brs.2009.03.007 20161507 PMC2797131

[B21] Rossi S. Antal A. Bestmann S. Bikson M. Brewer C. Brockmöller J. . (2021). Clinical neurophysiology safety and recommendations for TMS use in healthy subjects and patient populations, with updates on training, ethical and regulatory issues : expert guidelines. Clin. Neurophysiol. 132, 269–306. doi: 10.1016/j.clinph.2020.10.003 33243615 PMC9094636

[B22] Stewart L. M. Walsh V. Rothwell J. C. (2001). Motor and phosphene thresholds: a transcranial magnetic stimulation correlation study. Neuropsychologia 39, 415–419. doi: 10.1016/S0028-3932(00)00130-5 11164880

