# Supplementary material for: Blue magnetophosphene perception under subthreshold magnetic stimulation in a human retinal degeneration model
Source: Front Neurosci. 2026 Sep 10;20:1893022. doi: 10.3389/fnins.2026.1893022 (PMC13601358; doi:10.3389/fnins.2026.1893022)
Supplement: Supplementary file 1 [file Data_Sheet_1.docx]

**SUPPLEMENTARY -** Blue Magnetophosphene Perception Under Subthreshold Magnetic Stimulation in a Human Retinal Degeneration Model

Omar Nawara, Eleonore Fresnel, Verena Juncal, Frank S Prato, Cindy Hutnik, and Nicolas Bouisset^*^
*(nbouisset@lawsonimaging.ca)*

# The Clinical Presentation

The subject is an 82-year-old Caucasian male and retired learned professional with significant visual impairment. The subject’s ocular history is significant for a macular dystrophy secondary to Stargardt disease (STGD), primary open angle glaucoma (POAG), pigment dispersion syndrome, myopia, and a prior nuclear sclerotic cataract extraction and intraocular lens (CEIOL) exchange.

The subject’s primary vision loss was consequence of an early and profound macular dystrophy, which was diagnosed clinically as STGD, an autosomal recessive inherited macular dystrophy caused by biallelic variants in the ABCA4 gene on chromosome 1 ^1,2^. As a result of this mutation, there is an accumulation of visual cycle kinetics byproducts in the retinal pigment epithelium (RPE) with subsequent photoreceptor and RPE death within the macula ^1^. The subject began to experience bilateral visual deterioration when he was in his second decade of life, including a loss of visual acuity, central vision loss, and colour vision dysfunction.

The subject’s past medical history includes hyperlipidemia and migraines. The subject’s medications include atorvastatin, aspirin, omega-3 fatty acids, vitamin D3, and coenzyme Q10. Additionally, the subject’s family history was notable for diabetes on the paternal side. Functionally, the subject lives alone and is largely independent in his activities of daily living.

In terms of clinical findings, the subject’s uncorrected visual acuity was counting fingers in each eye at his routine ophthalmic assessment and measured 20/800 at the most recent low-vision clinic ophthalmic assessment. The low-vision clinic assessment also identified severely reduced contrast sensitivity, and eccentric preferred retinal loci, typically seen in subjects with central macular scotomas ^3^. The subject’s intraocular pressure is stable and within pressure control targets, with the most recent measurement at 13 mmHg in each eye. A right relative afferent pupillary defect was documented. Prior examinations demonstrated a central corneal thickness (529 and 515 microns in the right and left eyes, respectively), bilateral Krukenberg spindles, and iris transillumination defects. Gonioscopy showed wide open angles to the ciliary body with moderate to heavy pigmentation. Such findings have been described as consistent with glaucomatous changes to the anterior segment ^4,5^. Fundoscopy revealed bilateral and profound chorioretinal atrophy in addition to cup to disc ratios were near 0.8, bilaterally with average scleral diameters.

Prior diagnostic investigations included visual fields testing, spectral domain optical coherence tomography (OCT), intravenous fluorescein angiography (IVFA, Figures S3), colour fundus photography (Figure S2), and IOL biometry. Prior investigations also included a *Humphrey Field Analyzer 24-2* visual field test (i.e., 54 points spanning measuring 24 and 30 degrees temporally and nasally, respectively; HFA 24-2). The HFA-24 identified generalized depression in both eyes with a more nasally dominant defect in the left eye. Subsequent visual field tests were not performed due to the subject’s inability to complete them. Prior OCT and IVFA analyses showed optic nerve rim thinning (approximate disc diameter of 1.4 mm) macular atrophy, ganglion cell layer thinning, loss of retinal nerve fibre layer, choroidal thinning, and fibrosis. Colour fundus photographs identified macular atrophy and fundus flecks (as shown in ***Figures S1, S2, S3***). These multimodal imaging features are consistent with classic features of STGD ^1,6^.^.^

Preoperative planning for IOL implantation included biometry, which identified axial lengths of 26.13 mm and 25.78 mm, for OD and OS, respectively. The corresponding optical powers for the planned monofocal IOLs were selected based on a plano target and were calculated to be 15.0D and 16.5D, in OD and OS, respectively. These biometry findings are compatible with the subject’s history of myopia.

Electroretinography (ERG) testing was conducted using full field and multifocal (mfERG) modalities, using the International Society for Clinical Electrophysiology of Vision (ISCEV) protocol ^7,8^. The ISCEV‑standard full‑field electroretinogram responses were not measurable above noise for rod‑weighted, mixed, and cone‑weighted conditions in both eyes, including 30 Hz flicker. ISCEV‑standard multifocal electroretinogram showed markedly reduced and delayed first negative peak to first positive peak complex (N1‑P1) responses across most eccentricities with some traces not measurable, consistent with severe bilateral retinal dysfunction involving both rod and cone pathways with pronounced macular involvement ^9,10^.

In terms of active interventions, the subject utilizes topical ophthalmic prostaglandin analogues (bimatoprost 0.01%) in both eyes to treat his POAG. Prior intraocular pressure lowering medications included topical the betablocker, timolol. Surgically, the subject has also received CEIOL exchange (Tecnis® ZCB00; 15.0D OD and 16.5D OS) and subsequent posterior Yttrium Aluminum Garnet (YAG) laser capsulotomies, in both eyes. Interestingly, following the subject’s CEIOL surgery, the subject persistently reported a generalized blue hue to perceived light (his cataracts were orange-brown tinted prior to extraction).

# References

1.	Fujinami, K. *et al.* Stargardt macular dystrophy and therapeutic approaches. *British Journal of Ophthalmology* bjo-2022-323071 (2023) doi:10.1136/bjo-2022-323071.

2.	Bianco, L. *et al.* Association Between Genotype and Phenotype Severity in *ABCA4*-Associated Retinopathy. *JAMA Ophthalmol.* 141, 826 (2023).

3.	Li, S., Deng, X. & Zhang, J. An Overview of Preferred Retinal Locus and Its Application in Biofeedback Training for Low-Vision Rehabilitation. *Semin. Ophthalmol.* 37, 142–152 (2021).

4.	Silvina, A., Varas, S. & Valerio, R. Krukenberg spindle and its implications. *Advances in Ophthalmology &amp; Visual System* 12, 81–85 (2022).

5.	Scuderi, G. *et al.* Pigment dispersion syndrome and pigmentary glaucoma: a review and update. *Int. Ophthalmol.* 39, 1651–1662 (2018).

6.	Heath Jeffery, R. C. & Chen, F. K. Stargardt disease: Multimodal imaging: A review. *Clinical &amp; Experimental Ophthalmology* 49, 498–515 (2021).

7.	Hoffmann, M. B. *et al.* ISCEV standard for clinical multifocal electroretinography (mfERG) (2021 update). *Documenta Ophthalmologica* 142, 5–16 (2021).

8.	Robson, A. G. *et al.* ISCEV Standard for full-field clinical electroretinography (2022 update). *Documenta Ophthalmologica* 144, 165–177 (2022).

9.	Chiang, T.-K. & Yu, M. Electrophysiological Evaluation of Macular Dystrophies. *J. Clin. Med.* 12, 1430 (2023).

10.	Sajovic, J., Meglič, A., Hawlina, M. & Fakin, A. Electroretinography as a Biomarker to Monitor the Progression of Stargardt Disease. *Int. J. Mol. Sci.* 23, 16161 (2022).

# Figures

**Figure S1. External ocular globe photographs.** Top: right eye. Bottom: left eye.

**Figure S2. Color fundus photographs**. Top: right eye. Bottom: left eye.

**A**

**B**

**C**

**D**

**F**

**E**

**Figure S3. Fluorescein angiography of the left eye. A**. Pre‑choroidal (00:00.984). **B.** Choroidal flush (00:09.248). **C**. Arterial/early AV (00:16.140). **D**. Venous (00:33.652). **E**. Recirculation (01:31.379). **F.** Late phase (03:20.136).
